# Graves’ hyperthyroidism induced pancytopenia, epilepsia and muscle weakness: A case report

**DOI:** 10.1097/MD.0000000000031042

**Published:** 2022-10-14

**Authors:** Bao Fu, Dinghong He, Zhengguang Geng, Xiaoyun Fu

**Affiliations:** a Department of Critical Care Medicine, Affiliated Hospital of Zunyi Medical University, Zunyi City, Guizhou Province, China; b Department of Critical Care Medicine of Jinsha County People’s Hospital, Bijie City, Guizhou Province, China.

**Keywords:** epilepsia, Graves’ hyperthyroidism, muscle weakness, pancytopenia

## Abstract

Pancytopenia and epilepsia are rare complications of Graves’ disease (GD). Muscle weakness is a physical sign of GD. It is extremely rare for GD patients to present 3 symptoms at the same time.

**Patient concerns:**

A 35-year-old female was admitted to hospital for dizziness for 1 day. The results of laboratory examination on admission showed pancytopenia and hypothyroidism. Her clinical manifestations include pancytopenia, epilepsy, and muscle weakness.

**Diagnosis:**

Graves’ hyperthyroidism.

**Interventions:**

She received endotracheal intubation, ventilator, antithyroid drugs, and hormone therapy.

**Outcome:**

The patient was discharged after treatment.

**Lesson:**

Severe complications caused by GD are rare and require antithyroid therapy. Although glucocorticoid is not recommended by the guidelines, it can effectively improve thrombocytopenia.

## 1. Introduction

Graves’ disease (GD) is an autoimmune thyroid syndrome and is the most common cause of hyperthyroidism.^[1]^ About 50% of GD patients lack characteristic clinical features, so they may be misdiagnosed. Neutropenia is more common in patients with GD, while anemia and thrombocytopenia occur less frequently. Pancytopenia secondary to GD is extremely rare. Here, we report a GD patient with pancytopenia and muscle weakness.

## 2. Case presentation

On December 26, 2020, a 35-year-old female was admitted to Affiliated Hospital of Zunyi Medical University for dizziness for 1 day. One day ago, the patient developed dizziness without disturbance of consciousness. There was no profound weight loss and fatigue in the last month. She reported that her family members have a history of hyperthyroidism. She did not use tobacco, alcohol, or illicit drugs, and was not on chronic medications.

On presentation, her temperature was 36.5 °C, heart rate 130 beats per minute, blood pressure 121/78 mm Hg, respiratory rate 22 breaths per minute, and oxygen saturation 97% while the patient was breathing ambient air. The physical examination also revealed tenderness on abdominal palpation, negative kidney percussion test, goiter (grade II, WHO 1960 classification), and pretibial myxedema without orbitopathy. The patient’s initial blood test results showed white blood cell level was 2.75 × 10^9^/L (normal range: 4–10 × 10^9^/L), the absolute value of neutrophils was 0.85 × 10^9^/L (normal range: 1.8–6.3 × 10^9^/L), the level of hemoglobin was 79.0g/L (normal range: 115–150 g/L) and platelet count was 16 × 10^9^/L (normal range: 100–300 × 10^9^/L). Thyroid function test showed thyroid-stimulating hormone (TSH) was 0.011 μIU/mL (reference range: 0.55–4.7), free triiodothyronine (FT3) was above 30.8 pmol/L (reference range: 2.77–6.31) free thyroxine (FT4) was 116.6 pmol/L (reference range: 10.45–24.38), thyroid peroxidase antibody (TPOAb) was above 600 IU/mL (reference range: 0–34), thyroglobulin antibody (ATG) was 2567.0 ng/mL (reference range: 3.5–77) and thyrotropin receptor antibody (TRAb) was 25.78 IU/L (reference range: <1.75).

The initial 12-lead electrocardiogram (ECG) showed atrial fibrillation. No obvious abnormality was found in head CT examination. Chest CT showed localized emphysema, enlarged heart, and enlarged double lobes of thyroid. The neck ultrasound examination showed enlarged diffuse goiter (the right leaf of the thyroid: 62 × 32 × 24 mm, the left leaf: 66 × 30 × 26 mm, thyroid isthmus:10 mm). On the second day of admission (12/27/2020), the patient developed epileptic convulsions, which lasted for about 2 minutes. No abnormality was found in head fMRI. Cerebrospinal fluid examination and intracranial pressure also showed normal. Bone marrow biopsy smear showed megakaryoplasia of bone marrow, the number of thromocytogenic megakaryocyte was less and platelet was rare. Antinuclear antibody and platelet antibody were negative. Serum iron, haptoglobin, ferritin, folic acid, and vitamin B12 were normal. Electrophoresis of hemoglobin showed that HbA was 89.0% (reference range: 96.8%–97.8%), HbA2 was 2.00% (reference range: 2.2%–3.2%) and HbF was 9.00% (reference range: 0–0.5%).

On December 26, Graves’ hyperthyroidism was diagnosed and she received methimazole (MMI) treatment (10 mg, Tid) and propranolol (10 mg, Tid). On the fourth day after admission, the patient suffered frequent convulsions and somnolence. The patient was transferred to ICU after tracheal intubation due to frequent convulsions on February 1, 2021. After transfer to ICU, remifentanil and midazolam were used for analgesia and sedation, and levetiracetam was used to control convulsions. The convulsions disappeared on the fourth day after ICU admission. The sedative was stopped on the fourth day after entering the ICU, but the patient remained in a light coma. On the 10th day of ICU admission, the patient could open his eyes when calling, but the muscle strength of his limbs was grade I. On the 22nd day of ICU admission, the patient was awake and the muscle strength of his limbs recovered to grade IV.

**Figure 1.**
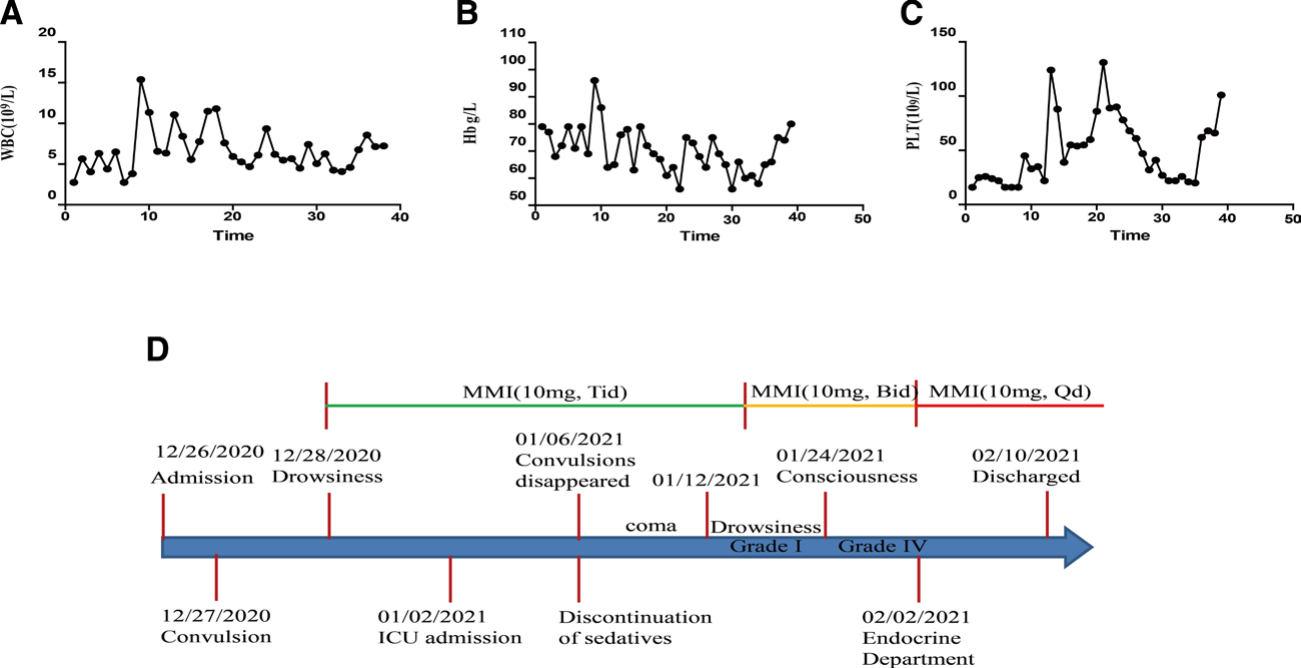
The changes of white blood cell count (A), hemoglobin (B) and platelet count (C). The treatment course of the patients (D).

After treatment with antithyroid drugs, white blood cells rose to normal level rapidly, but thrombocytopenia did not improve significantly (Fig. 1A and B). For thrombocytopenia, the patient received recombinant human thrombopoietin (rhTPO) treatment, but the platelet level was still unstable. The patient was given methylprednisolone 80 mg once a day, which was reduced to 40 mg once a day after three days. After methylprednisolone treatment, platelet returned to normal level very quickly (Fig. 1C). Methimazole 10 mg TID was reduced to 10 mg bid after 20 days treatment. On the 22nd day of ICU admission, the patient was extubated. On the same day, thyroid function test showed that TSH was 0.011 μIU/mL, FT3 7.4 pmol/L, FT4 18.4 pmol/L. On February 10, 2021, the patient was transferred to the Department of Endocrinology for further treatment. The oral dose of methimazole was reduced to 10 mg, Qd. Eight days after transferring to the Department of Endocrinology, the patient recovered and discharged (Fig. 1D). The mechanism of leukopenia is also poorly understood.

## 3. Discussion and Conclusions

The manifestations of GD depend on the patient’s age at the onset of hyperthyroidism, the severity and the duration of hyperthyroidism.^[2]^ The onset age of this patient was 35 years old, but the clinical symptoms were very serious. The patient developed Graves’ hyperthyroidism-related pancytopenia, epilepsy and muscle weakness caused by hyperthyroidism. The patient recovered after receiving antithyroid drugs. Such case is extremely rare and has not been reported in previous literatures.

Pancytopenia is a rare complication of GD, which indicates a serious clinical condition. The antithyroid drugs are among the main drugs potentially inducing pancytopenia, while the patient did not receive antithyroid drugs before admission. GD related pancytopenia can occur at all ages and the median age is less than 46 years (interquartile range 32–56 years), which is more frequently in women.^[3]^ There is an association between thyrotoxicosis and pancytopenia, although the underlying physiopathology is unclear. The reduced production of hematopoietic cells from the bone marrow and increased destruction or sequestration of mature hematopoietic cells may be the two main paths leading to pancytopenia.^[4]^ Anemia may be caused by exaggerated consumption of folic acid by iron, folic acid, and vitamin B12, while the levels of these indicators in this patient were within the normal range. Peripheral hemolysis was ruled out by normal values of indirect bilirubin, haptoglobin, and negative Coombs of the patient. Therefore, there may be other underlying physiopathology of anemia in this patient. The mechanisms of leukopenia are also poorly understood. A reduced marrow granulocyte reserve and the hypothesis of immunologic destruction mechanisms may lead leukopenia.^[4]^ A previus study showed significant correlations between T3 levels and absolute neutrophil counts, TSH levels and absolute CD4 + counts, T4 levels and absolute CD4 + counts.^[5]^ In some cases, antiplatelet antibodies have been found in the serum of patients with GD.^[6]^ However, the serum antiplatelet antibody was negative in the patient. This patient’s thrombocytopenia may be related to the decrease of thromocytogenic megakaryocyte caused by GD. We found that antithyroid drugs improved pancytopenia to some extent in the patient, but glucocorticoid had a better effect on thrombocytopenia, although the guidelines did not recommend it.

GD induced epilepsy is very rare and the mechanism is unclear. The patient had frequent epileptic seizures. Levecetam and antithyroid drugs effectively controlled the epileptic symptoms. Muscle weakness is a physical sign of hyperthyroidism.^[1]^ The reduction in muscle strength has been reported in previous studies.^[7,8]^ Lower levels of 25-hydroxyvitamin D (25(OH)D) and vitamin D deficiency were associated with low physical performance muscle weakness.^[9–11]^ (25(OH)D) level of the patient was lower than the reference range (13.77 ng/mL, reference range > 30 ng/mL). GD is associated with lower levels of 25(OH)D.^[12–15]^

In conclusion, pancytopenia and epilepsia are rare complications of Graves’ disease. Muscle weakness is a physical sign of Graves’ disease. It is extremely rare for GD patients to present three symptoms at the same time. Antithyroid drugs can be used to treat both pancytopenia and GD.

## Authors’ contributions

ZGG and DHH collected clinical datas. BF was responsible for writing manuscript. XF revised the manuscript. All authors have read and approved the manuscript.

**Conceptualization:** Dinghong He, Xiaoyun Fu.

**Formal analysis:** Dinghong He.

**Supervision:** Zhengguang Geng.

**Writing – original draft:** Bao Fu.

**Writing – review & editing:** Bao Fu.
